# The effect of curve running on distal limb kinematics in the Thoroughbred racehorse

**DOI:** 10.1371/journal.pone.0244105

**Published:** 2020-12-29

**Authors:** Rebecca S. V. Parkes, Thilo Pfau, Renate Weller, Thomas H. Witte

**Affiliations:** 1 Department of Veterinary Clinical Sciences, City University of Hong Kong, Kowloon, Hong Kong; 2 Department of Clinical Science and Services & Structure and Motion Lab, Royal Veterinary College, South Mimms, Hertfordshire, United Kingdom; Massey University, NEW ZEALAND

## Abstract

During racing, injury is more likely to occur on a bend than on a straight segment of track. This study aimed to quantify the effects of galloping at training speeds on large radius curves on stride parameters and limb lean angle in order to assess estimated consequences for limb loading. Seven Thoroughbred horses were equipped with a sacrum-mounted inertial measurement unit with an integrated GPS, two hoof-mounted accelerometers and retro-reflective markers on the forelimbs. Horses galloped 2–4 circuits anticlockwise around an oval track and were filmed at 120 frames per second using an array of ten cameras. Speed and curve radius were derived from GPS data and used to estimate the centripetal acceleration necessary to navigate the curve. Stride, stance and swing durations and duty factor (DF) were derived from accelerometer data. Limb markers were tracked and whole limb and third metacarpus (MCIII) angles were calculated. Data were analysed using mixed effects models with a significance level of p < 0.05. For horses galloping on the correct lead, DF was higher for the inside (lead) leg on the straight and on the curve. For horses galloping on the incorrect lead, there was no difference in DF between inside and outside legs on the straight or on the curve. DF decreased by 0.61% of DF with each 1 m s^-2^ increase in centripetal acceleration (p < 0.001). Whole limb inclination angle increased by 1.5° per 1 m s^-1^ increase in speed (p = 0.002). Limb lean angles increase as predicted, and lead limb function mirrors the functional requirements for curve running. A more comprehensive understanding of the effects of lean and torque on the distal limb is required to understand injury mechanisms.

## Introduction

In nature, the negotiation of curves is important during locomotion, for example during predator-prey interactions where speed alone may be insufficient to allow escape or a successful hunt [1]. Domesticated horses negotiate curves and bends frequently, both in racing and other disciplines, with horses often training and racing on oval tracks in the USA, Australia and elsewhere. It has been suggested that increased limb force associated with galloping on a curve may be associated with increased injury risk [2]. Sharp turns increase the risk of ligamentous injury in the foot [3,4] and during racing catastrophic injury is more likely to happen on a curve than on a straight segment of track [5,6]. Despite a high incidence of musculoskeletal injuries during training [7–10], no study has yet assessed the changes in gait associated with galloping at training speeds on a curved track.

Running on a curve leads to an increased force perpendicular to the direction of motion to effect a change in the direction of the centre of mass [2,11]. This centripetal force increases with higher speed and a smaller radius of the curve. Maximum running speed in human runners [11], and in Thoroughbred racehorses [2] is slower on a curve than in a straight line. Humans [12] and mice [1] maintain stride frequency and increase foot contact time when negotiating curves. In humans, the inside limb produces a smaller peak vertical ground reaction force (vGRF) than the outside limb, and smaller radii lead to a smaller peak vGRF than larger radii, with a shorter step length at similar speeds [11] and as vGRF decreases, horizontal GRF (hGRF) increases [11]. However, greyhounds do not increase foot contact timings, and consequently experience a 65% increase in limb forces as they negotiate a bend [13]. The inside and outside forelimbs experience a similar increase in limb force, but the leading hindlimb experiences a greater increase in force on the bend compared with the nonlead hind limb [13]. On tighter bends other breeds of dog follow a similar pattern to other species and increase duty factor with decreasing curve radius [14].

Horses prolong stride duration on a 15 m circle compared with a straight line [4]. The inside limb experiences a higher duty factor [4] and therefore a lower peak vertical ground reaction force (vGRF) [15] than the outside limb when cantering on a flat 10 m circle and when trotting on a 4m circle [16]. At slower speeds, the impulse generated by inside and outside limbs does not differ [17]. Horses exhibit body lean into the centre of a curve at walk [4], trot [4,18,19] and canter [19] on bends. Lean is greater on flat than banked curves in walk, trot and canter, with horses sometimes leaning outwards on banked curves with respect to the bearing surface [4]. On flat surfaces, the degree of lean varies between individual horses, and does not always accurately reflect predicted leans [19], with some degree of individual variation and laterality [20]. The degree of lean is generally less than the amount predicted by mathematical predictions [19], which may be due to bend along the long axis of the trunk. In addition to body lean, limb angle changes to adapt to curved locomotion, with greater third metacarpal bone (MCIII) inclination seen on flat curves compared with banked curves, and the inside limb exhibiting a greater inclination than the outside limb [4]. Third metatarsal bone (MTIII) inclination follows a similar pattern, although angles are lower than for MCIII [4]. It is likely that these changes in limb angle are to optimise the alignment of the resultant GRF with the long axis of the limb [11]. Furthermore, horses appear to adapt their gait when running on a curve with time, suggesting that there is some adaptation with training [21]. However, given the likely force limitation on top speed on large radius curves [2] and the increase in injury rate seen in horses on a curve [5,6] a better understanding of adaptation to curve running would be beneficial.

The objective of this study was to assess the effects of galloping on a curve on stance duration, swing duration, stride duration and forelimb inclination on bends of low curvature (i.e., of a size that might be seen in race training) at training speeds and to compare actual limb lean to predicted limb inclination angles.

It was hypothesised that stance duration and duty factor would be greater during curve running than during straight line running in order to manage force limits on curved locomotion. The secondary hypothesis was that limb inclination would be greater for the inside limb then the outside limb on the curve.

## Materials and methods

The Clinical Research and Ethical Review Board at the Royal Veterinary College granted ethics approval for this study (URN 2013 1232).

### Data collection

Seven Thoroughbred horses were convenience sampled from a population that had retired from racing but were still undertaking regular gallop training. Informed verbal consent was obtained from the horse’s owners before participation in the trials.

Each horse was equipped with two biaxial hoof-mounted ±50 g accelerometers (ADXL150, Analog Devices, Norwood, MA, USA) logging at 1000 Hz to an internal SD card. The accelerometers were placed on the left fore (LF) and right fore (RF) hooves, attached over the dorsal midline of the hoof with hot glue. Each horse also had one Global Positioning System-Inertial Measurement Unit (GPS-IMU; in- house design; Structure and Motion Laboratory, Royal Veterinary College) logging at 300 Hz to an internal SD card placed over the most caudal palpable dorsal spinous process, attached using a woven elastic self-adhesive porous foam plaster (Animal Polster, Snøgg, Norway). Three circular retro-reflective markers were placed on the midline of each forelimb, one on the proximal antebrachium, one on the proximal metacarpus and one on the distal metacarpus, attached using double-sided tape.

An array of ten high-speed cameras (GoPro Hero 3, San Mateo, CA, USA) was set up attached to the running rails on the outside of an oval gallop, with one camera facing down the straight side of the gallop and nine cameras at five metre intervals along the curved side of the oval gallop, filming horses galloping towards the cameras on the curve. Cameras were set at a resolution of 1280 x 720 pixels and recorded at 120 frames per second.

Accelerometers, IMUs and cameras were manually synchronized. The hoof-mounted accelerometers and sacrum-mounted GPS-IMU were taped together and tapped five times before attaching them to the horse to allow manual synchronisation of the accelerometers to GPS time following the experiment using the acceleration peaks generated. A further GPS-IMU was attached to a clapper board and several taps were filmed by the GoPro cameras to allow synchronisation of the GoPro data to GPS time. A spirit level, accurate to 0.1°, was used for alignment of the GoPro cameras to the vertical.

Horses performed a routine warm-up at walk and trot according to their usual training regimen before galloping in an anticlockwise direction around the oval synthetic track for 2–4 circuits. Therefore the left limbs were on the inside of the curve, and the right on the outside and the left forelimb as lead leg is the ‘correct’ lead. For clarity, the left forelimb will be referred to as the inside limb and the right forelimb as the outside limb.

The track was regularly maintained with daily harrowing and consisted of waxed sand. Trials took place in the early morning when the track was freshly harrowed. The track was flat, with no banking at the curves. Horses galloped between 960m and 1920m in total, according to their usual training regimen. Riders were instructed not to ask the horses to change legs but to allow the horses to gallop how they preferred.

### Data analysis

Data were analysed using custom-written software in MATLAB (MATLAB, R2012b, The Mathworks, Natick, MA, USA). The hoof-mounted accelerometer data were down-sampled to 300 Hz and synchronised with sacrum-mounted GPS-IMU data. Data for periods when the horses were travelling at greater than 6 m s^-1^ on the oval track were identified using GPS data. Speed was calculated from GPS data. Stride duration and stance duration for the forelimbs were derived from accelerometer data using acceleration peaks for foot on and foot off as described by Witte et al. [15]. The hoof contact sequence allowed identification of the lead limb. Curve radius was calculated using the following formulae:

$$\omega =\frac{\Delta \theta }{\Delta t}$$ (1)

$$ri=\frac{v}{\omega }$$

Curve radius was then used to calculate centripetal acceleration as below:

$$a_{c}=\frac{v^{2}}{r}$$

Where *ω* is angular velocity of the horse on its curved path, *Δθ* is change in heading from GPS (in radians) over one stride, *Δt* is stride duration, *r*_i_ is effective radius (i.e., radius derived from change in heading) and *v* is speed. The change in angle was measured by the dorsally mounted IMU, and the speed derived from GPS data.

Curve radius was used to categorise data as ‘curve’ or ‘straight’, with curves larger than 200 m radius being defined as ‘straight’. Video data was synchronised with GPS time. Limb markers were tracked during mid-stance for strides where the horse was visually judged to be directly facing the camera using two dimensional tracking software in MATLAB [22], and whole limb angle and metacarpus angle to the vertical were calculated based on two-dimensional coordinates for each marker and averaged for each stride. Strides where the horse was not judged to be directly facing the camera were discarded. Positive values indicate a lean towards the inside of the bend, and negative values indicate a lean towards the outside of the bend.

Predicted whole body lean was calculated from speed and curve radius as described by Brocklehurst et al [19] and the difference in predicted lean and measured limb inclination angle was calculated by subtracting the measured angle from the predicted angle.

Angular velocity plotted was then against speed for each stride collected, and compred to the cutoff for speed at a given angular velocity based on a coefficient of friction (μ) of 0.6, as described by Tan & Wilson [2].

### Statistical analysis

Data for stride duration, stance duration, swing duration and duty factor were analysed using a mixed effects model (p < 0.05) with horse as a random effect and speed, limb, curve category and lead leg as fixed effects to establish differences in stride parameters when galloping on the straight and curved sections of the track. Straight segments of track may be calculated as having an infinite radius when calculated from change in heading, and as such curve category (‘straight’ or ‘curve’) was used as a discrete variable in the mixed effects model to make analysis possible. Further mixed effects models with horse as a random effect and speed, limb, curve category and lead leg as fixed effects were used to assess changes in whole limb angle and in MCIII angle. The effects of centripetal acceleration on duty factor, whole limb angle and MCIII angle were assessed using linear regression as increased centripetal acceleration increases effective body mass and therefore may limit speed due to an increase in force on the limbs [2]. Centripetal acceleration could not be assessed within a mixed model with speed and curve category, as these factors directly affect centripetal acceleration. As many horses will choose to canter or gallop on a curve with the inside fore as the ‘lead leg’, there is likely to be an interaction between lead leg and curve, and due to the different functions of lead and nonlead legs in the gallop, there is likely to be an interaction between lead and leg categories. Non-significant effects were excluded from the final models and a pairwise Tukey post-hoc comparison was used to assess interactions between leg, lead leg and curve category. Residuals for all models were normally distributed and followed a non-biased homoscedastic pattern.

Predicted and measured angles for MCIII and the whole limb on the straight and on the curve for inside and outside legs did not all follow a normal distribution as determined using a Shapiro-Wilk test for normality. Differences between predicted and measured angles for MCIII and the whole limb for inside and outside legs on the curve and on the straight were compared using a Mann-Whitney U test. Differences between MCIII and whole limb angle were also compared using a Mann-Whitney U test. All statistical analysis was performed in R (R Project for Statistical Computing, Vienna, Austria).

## Results

**Fig 1:**
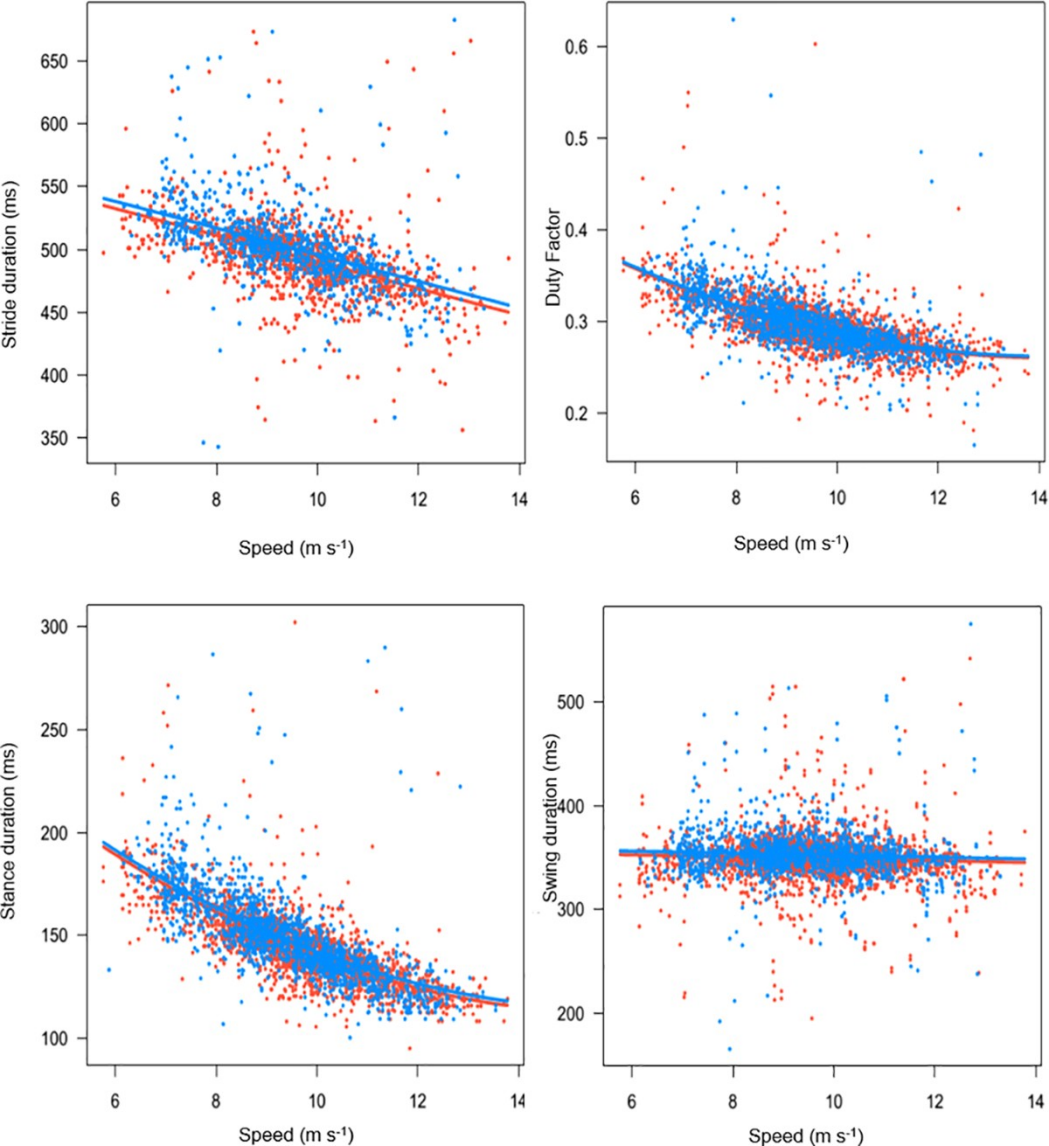
Relationship between speed and stride duration, stance duration, swing duration and duty factor. Red points are inside (left) forelimb and blue points are outside (right) forelimb.

A total of 1931 strides were used for analysis of stance duration, of which 1130 were on a curve and 801 were on the straight, with 359 right lead strides (outside leg) and 1572 left lead strides (inside leg). Of these, 164 right lead strides were on the straight and 195 on the curve Eighty strides were used to assess inside limb angle and 95 strides were used to assess outside limb angle from straight and curved sections of the track. One horse was excluded from limb angle assessment due to poor quality video data. The average speed across straight and curved data was 9.5 m s^-1^, range 6.0 m s^-1^ to 13.7 m s^-1^, and the average stride duration across straight and curved data was 498.8 ms ± (s.d.) 34.0 ms. The effects of speed on stride duration, stance duration, swing duration and duty factor are displayed in Fig 1. The average whole limb inclination in relation to the vertical was 9.4°± 10.6 and limb inclination angle ranged from -20.6° to 36.9°.

### Changes to stride parameters on a curve

Stride duration showed a linear decrease of 10.60 ms per 1 m s^-1^ increase in speed (p < 0.001). Stride duration on the correct lead was 3.97 ms reduced compared to on the incorrect lead (p = 0.006) and stride duration on a curve was 5.72 ms longer than on the straight (p < 0.001), without accounting for speed.

**Fig 2:**
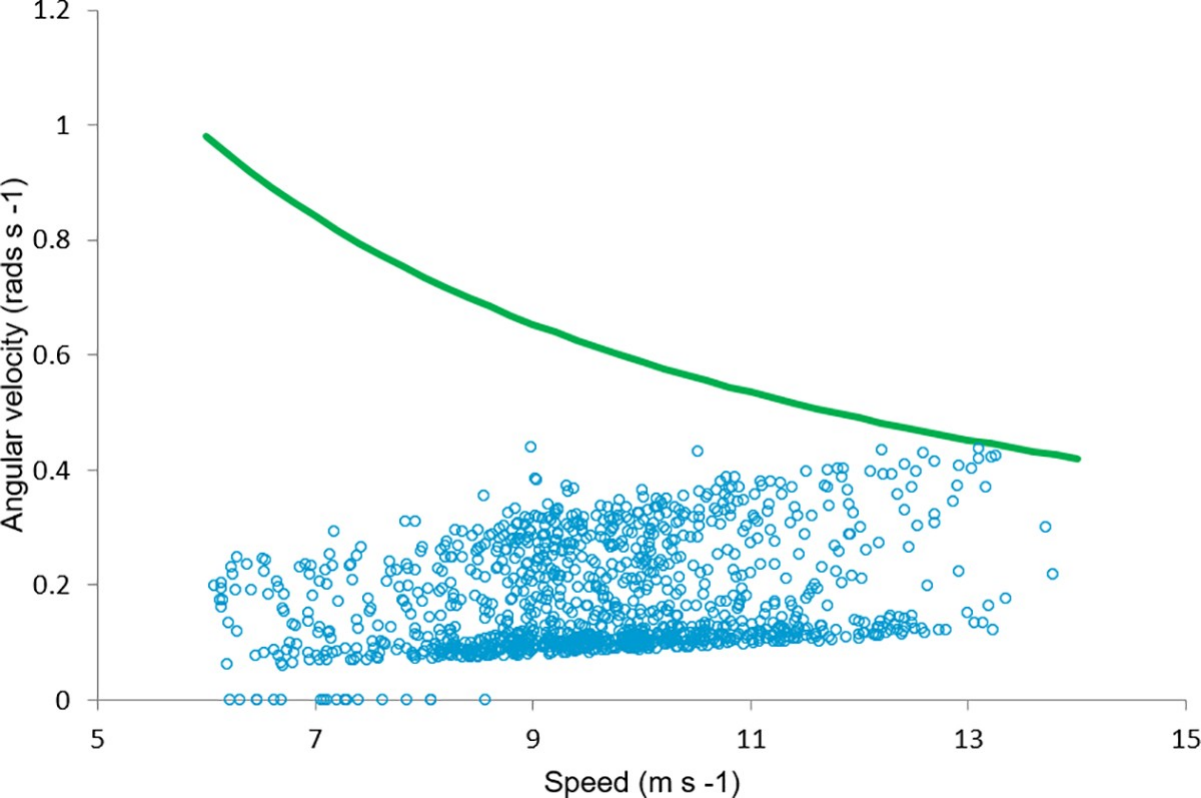
Friction affects smaller turns at higher speeds. Angular velocity plotted against speed for each stride collected. The green line represents the cutoff for speed at a given angular velocity based on a coefficient of friction (μ) of 0.6, as described by Tan & Wilson [2].

When angular velocity was plotted against speed and compared to the cutoff for maximum speed as limited by friction, based on a coefficient of friction (μ) of 0.6, as described by Tan & Wilson [2], it appears that some horses may have approached the limits fo frictin at around 14ms^-1.^ (Fig 2).

Horses showed an overall decrease in stance duration with increasing speed (p < 0.001). There was a non-linear pattern of stance duration change with increasing speed, with the linear and quadratic coefficients being -25.8 ms (p < 0.001) per 1 m s^-1^ increase in speed and 0.83 ms^2^ (p < 0.001) per 1 m s^-1^ increase in speed respectively. There was a non-linear pattern of stance duration change with increasing speed. For a horse galloping on the correct lead, the inside leg had a longer stance duration than the outside leg both on a curve and on the straight. For a horse galloping on the incorrect lead, there was no difference between inside and outside leg stance time on the curve, and the inside (non-lead) leg had a longer stance duration on the straight. For both correct and incorrect lead strides, both inside and outside legs had a longer stance duration on the straight than on a curve (Table 1).

**Table 1:** Results from mixed models.

Stride Parameter	Effect	Category	Estimate	Standard error	p-value
**Stride Duration (ms)**	Speed		-10.6	5.57	<0.001
	Curve	Curve	0		
		Straight	5.57	0.95	<0.001
	Lead Leg	Outside	0		
		Inside	-3.96	1.45	0.006
**Stance duration (ms)**	Speed		-25.8	1.646	<0.001
	Speed^2^		0.83	0.09	<0.001
Correct Lead	RF Curve#	LF Curve#	-3.36	1.38	0.015
	LF Straight	LF Curve	5.03	1.44	<0.001
	RF Straight	RF Curve	4.50	1.44	0.002
	RF Straight	LF Straight	-3.90	1.49	0.009
Incorrect lead	RF Curve	LF Curve	0.30	0.63	0.635
	LF Straight	LF Curve	3.87	0.70	<0.001
	RF Straight	RF Curve	1.81	0.70	0.010
	RF Straight	LF Straight	-1.76	0.77	0.022
**Swing duration (ms)**	Speed		-1.00	0.32	0.002
Correct Lead	RF Curve	LF Curve	4.83	2.61	0.065
	LF Straight	LF Curve	5.25	2.73	0.055
	RF Straight	RF Curve	4.41	2.72	0.105
	RF Straight	LF Straight	4.00	2.83	0.158
Incorrect Lead	RF Curve	LF Curve	-0.038	1.19	0.974
	LF Straight	LF Curve	2.63	1.33	0.048
	RF Straight	RF Curve	3.73	1.33	0.005
	RF Straight	LF Straight	1.06	1.45	0.465
**Duty Factor (%)**	Speed		4.09%	0.29%	<0.001
	Speed ^2^		0.14%	0.02%	<0.001
Correct lead	RF Curve	LF Curve	-0.73%	0.24%	0.003
	LF Straight	LF Curve	0.25%	0.26%	0.328
	RF Straight	RF Curve	0.27%	0.25%	0.292
	RF Straight	LF Straight	-0.72%	0.26%	0.007
Incorrect lead	RF Straight	RF Curve	0.03%	0.12%	0.794
	RF Curve	LF Curve	0.08%	0.11%	0.450
	LF Straight	LF Curve	0.24%	0.12%	0.057
	RF Straight	LF Straight	-0.12%	0.14%	0.376
	Centripetal acceleration		-0.61%	0.06%	<0.001

This table shows the effects of speed, curve, leg, lead leg and centripetal acceleration on stride, stance and swing duration and duty factor. LF = left foreleg, RF = right foreleg. Speed^2^ represents the non-linear change in stance and duty factor seen in relation to speed. Reference categories are noted with an estimate of 0. To understand the post-hoc comparison, compare the ‘Effect’ column compared to the ‘Category’ column, so for example, the LF stance duration on a curve is longer than the RF stance duration on a curve (#).

Swing duration reduced by 1.0 ms (p = 0.002) per 1 m s^-1^ increase in speed. For horses galloping on the correct lead, there was no difference in swing duration between inside or outside legs on a curve or on the straight. For horses galloping on the incorrect lead, the inside leg and the outside leg both had a longer swing duration on the straight than on a curve (Table 1).

**Fig 3:**
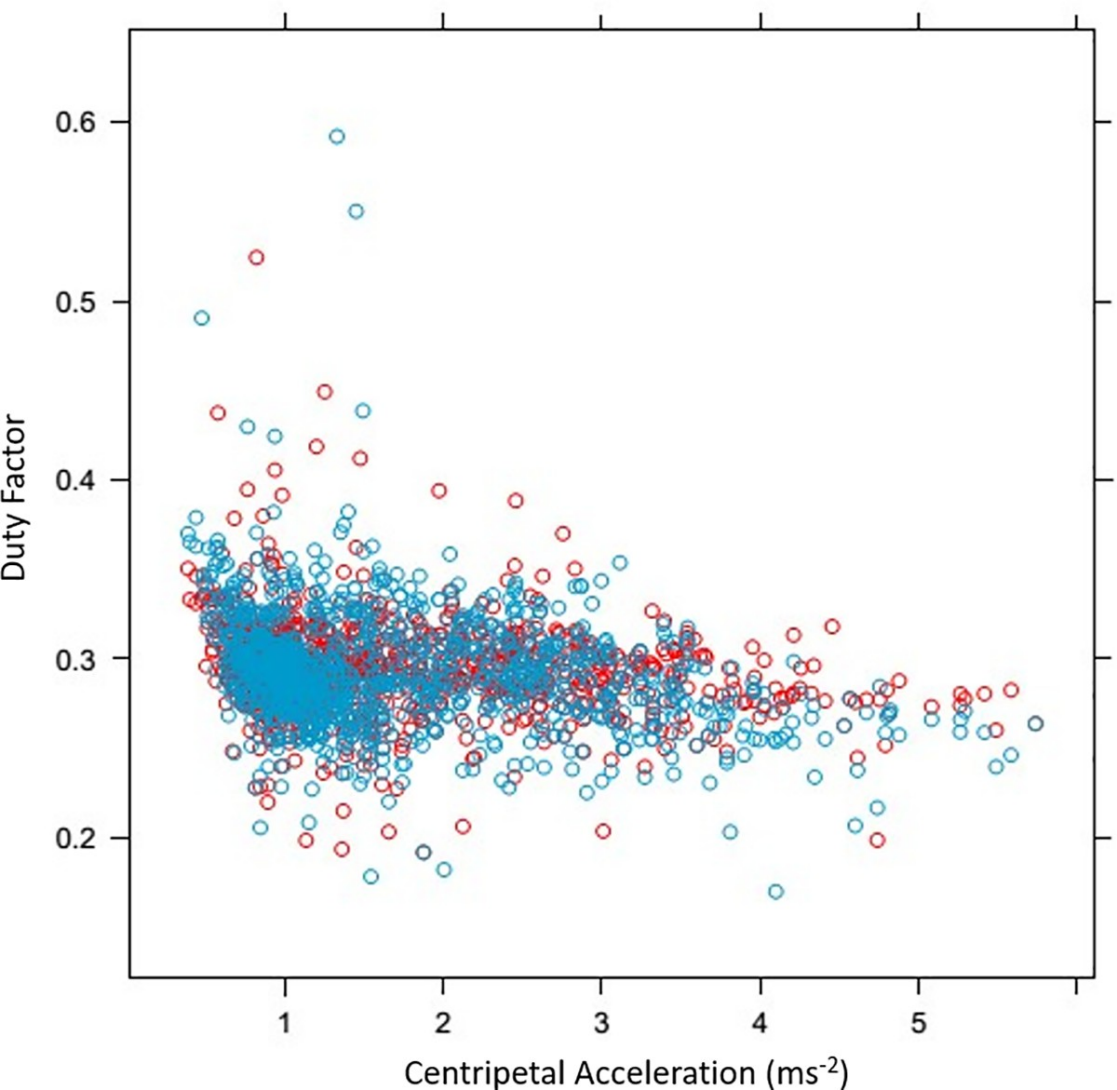
Relationship between duty factor (DF) and centripetal acceleration (as calculated from circle radius and speed). Red points are inside (left) forelimb and blue points are outside (right) forelimb. DF decreased by 0.61% with each 1 m s^-2^ increase in centripetal acceleration (p < 0.001).

Duty factor (DF) showed a non-linear pattern in relation to speed, decreasing with increasing speed, with the linear and quadratic coefficients being -4.1% (p < 0.001) per 1 m s^-1^ increase in speed and -0.14% (p < 0.001) per m s^-1^ increase in speed respectively. For horses galloping on the correct lead, DF was higher for the inside leg on the straight and on the curve. For horses galloping on the incorrect lead, there was no difference in DF between inside and outside legs on the straight or on the curve. DF decreased by 0.61% with each 1 m s^-2^ increase in centripetal acceleration (p < 0.001) (Fig 3).

### The effect of curve running on limb inclination angle

**Fig 4:**
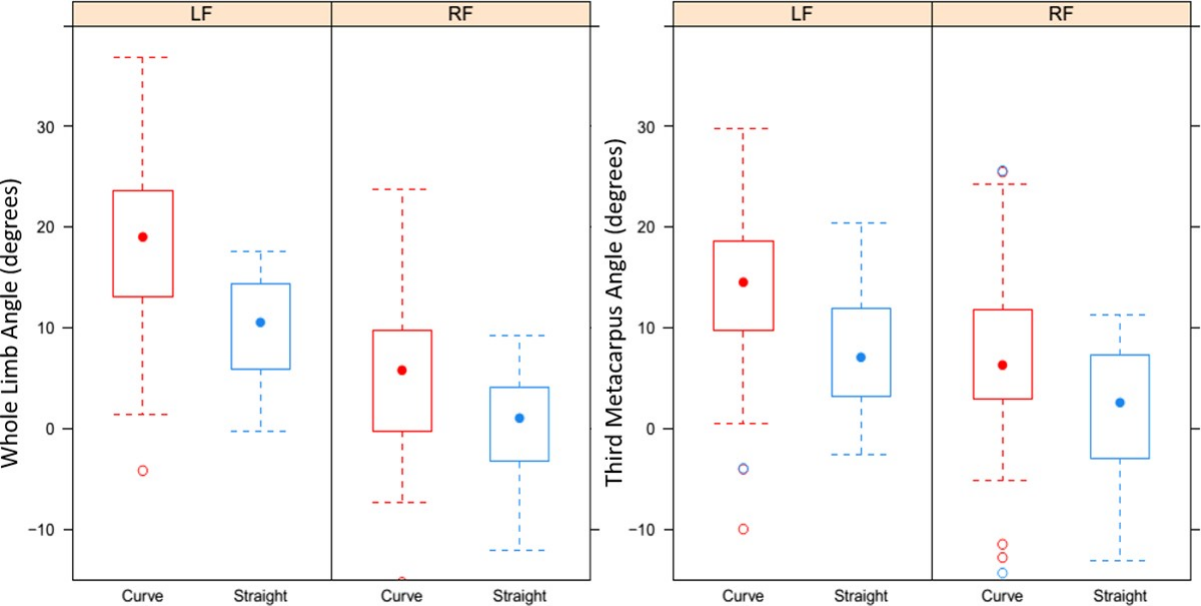
Effect of curve running on limb inclination. Boxplots show the mean (dot) and quartiles (box) of absolute angles for the whole limb and third metacarpus a curve (red) and on the straight (blue) for inside (LF) and outside (RF) forelimbs.

The average limb inclination angles for inside and outside legs on a curve and on the straight are shown in Table 2. A positive angle signifies an inclination towards the inside of the curve, and a negative angle an inclination towards the outside of the curve. There was in increase in whole limb inclination of 1.5° per 1 m s^-1^ increase in speed (p = 0.002). Lead leg did not affect limb inclination (p = 0.57). On the straight, the inside leg was at an average of a 9.8° greater angle than the outside leg (p < 0.001), and on a curve the inside leg was at a 14.1° greater angle than the outside leg (p < 0.001). The inside leg was at an 8.26° greater angle on a curve than on the straight (p < 0.001), and the outside leg was at a 3.97° greater angle on a curve than on the straight (p < 0.001) (Fig 4). There was an increase in MCIII angle of 4.4° per 1 m s^-2^ increase in centripetal acceleration (p < 0.001). Third metacarpus (MCIII) angle increased in relation to the vertical by 1.31° with each 1 m s^-1^ increase in speed (p = 0.008). On the straight, the inside leg (MCIII) was at a 5.92° greater angle than the outside leg (p = 0.01) and on a curve, the inside leg was at a 7.18° greater angle than the outside leg (p<0.001). The inside leg (MCIII) was at a 5.81° greater angle on a curve compared to on the straight (p = 0.005), and the outside leg was at a 4.55° greater angle on a curve than on the straight. There was no effect of lead on MCIII angle (p = 0.45). There was an increase in MCIII angle of 3.96° per 1 m s^-2^ increase in centripetal acceleration (p < 0.001). Full results for limb inclination models are shown in Table 3.

**Table 2:** Limb inclination angles on the curve and on the straight.

Curve category	Leg	Speed (m s ^-1^)	Measured angle	Predicted angle	Difference	p- value
**MCIII**						
Curve	Inside	9.60 ±1.37	13.70 ±7.85	12.60 ±5.11	-1.10 ±6.36	0.18
	Outside	9.62 ±1.39	6.61 ±8.39	12.60 ±5.74	6.00 ±7.91	<0.001
Straight	Inside	9.29 ±1.11	7.92 ±6.09	2.50 ±1.34	-5.42 ±5.75	0.001
	Outside	9.46 ±1.15	2.64 ±6.81	2.84 ±1.63	0.21 ±6.67	0.89
**Whole Leg**						
Curve	Inside	9.60 ±1.37	18.62 ±8.37	12.60 ±5.11	6.01 ±6.86	<0.001
	Outside	9.62 ±1.39	4.57 ±8.53	12.60 ±5.74	8.04 ±7.60	<0.001
Straight	Inside	9.29 ±1.11	10.04 ±5.96	2.50 ±1.34	-7.54 ±5.51	<0.001
	Outside	9.46 ±1.15	1.14 ±6.29	2.84 ±1.63	1.71 ±5.88	0.21

Limb inclination angles. Mean ± s.d. values for speed, measured angle, predicted angle and difference for inside and outside legs of Thoroughbred horses galloping on a curve and on the straight. Values for MCIII and whole limb angle are shown separately. p–values indicate if the difference between predicted and measured angles is significant as determined by a paired t-test.

**Table 3:** Results from mixed models.

**Effect**	**Category**	**Whole limb angle**		
		**Estimate**	**Standard error**	**p-value**
Speed		1.48	0.48	0.03
Interaction between curve and leg	LF Curve	0		
	RF Curve	-14.11	-10.51	<0.001
	LF Straight	-8.26	-3.92	0.001
	RF Straight	-18.08	-9.16	<0.001
Lead	Incorrect	0		
	Correct	0.97	1.67	0.57
Centripetal acceleration		4.40	0.63	<0.001
**Effect**	**Category**	**MCIII Angle**		
		**Estimate**	**Standard error**	**p-value**
Speed		1.31	0.49	0.008
Interaction between curve and leg	LF Curve	0		
	RF Curve	-7.19	1.29	<0.001
	LF Straight	-5.81	2.06	0.005
	RF Straight	-11.73	1.94	<0.001
Lead	Incorrect	0		
	Correct	1.33	1.74	0.45
Centripetal acceleration		3.96	0.62	<0.001

This table shows the effects of speed, curve, leg, lead leg and centripetal acceleration on whole limb and MCIII angle on Thoroughbred horses galloping. LF = left forelimb, RF = right forelimb. To understand the post-hoc comparison, compare the ‘Effect’ column compared to the ‘Category’ column.

For the inside leg, inclination predictions underestimated MCIII angle for the straight (p = 0.001) but did not significantly underestimate lean on a curve (p = 0.18). For whole inside leg inclination, predicted values overestimated limb angle for the curve (p < 0.001) and underestimated for the straight (p = 0.001). For the outside leg, predictions overestimated whole leg (p <0.001) and MCIII inclination on the curve (p<0.001).

Across all speeds, the mean whole limb angle on a curve for the inside limb was 4.46° greater than MCIII angle for the inside limb on a curve (p = 0.0008). There was no difference between whole and MCIII limb inclination angle for the outside limb on a curve or for either limb on the straight.

## Discussion

This study shows the changes in stride parameters and limb inclination angle in horses when galloping at training speeds on a large curve when compared to running on the straight. As hypothesised, both whole limb angle and MCIII angle increased with increasing speed, increased on a curve and consequently lean increased with increasing centripetal acceleration.

Duty factor is higher for the inside leg on the straight and on the curve when the horse is on the correct lead for a left-hand bend, but no difference is seen when the horse is on the incorrect lead. The correct lead is likely to result in a lower peak vertical GRF for the inside leg [23] and is consistent with previous studies showing that at a slow gallop, the peak vertical ground reaction force experienced by the nonlead limb is higher than for the lead limb [24,25], and that horses cantering on curves of small radius have a longer stance duration for the inside limb [4]. Depending on speed, the outside limb should generate more force than the inside limb [4,11], although the increase in impulse only applies to vertical, not horizontal forces [16]. We suggest that here the lack of a difference between inside and outside limbs seen when the horse is on the right (‘incorrect’) lead is due to a mismatch between the functions of the inside and outside legs on a bend and the functions of the lead and nonlead legs in a gallop. Given that horses usually gallop with the lead limb as the inside leg on a bend, there is likely to be an advantage to matching biomechanical functions, as the nonlead limb generates a greater force than the lead limb [15,26]. It is also possible that an increase in duty factor, and therefore a decrease in peak vertical force [15] may reduce injury risk to the horse. It is not known if galloping on the wrong lead around a bend at high speeds contributes to increased injury risk. However, as many horses display a preference for the right lead [27], the choice of the ‘incorrect’ lead could simply be an indication of this preference. Furthermore, the Thoroughbreds assessed here has all trained in different locations previously, and may have developed a preference for the right over the left lead prior to this.

The decrease in duty factor on a curve and with increasing centripetal acceleration is in contradiction to what would be expected if horses are minimising force, but the speeds in this study do not represent those seen during maximal performance when racing on a curve [2]. Note that the accuracy of centripetal acceleration here is reliant upon the accuracy of the GPS data logged, which may be affected by factors such as the number of available satellites and any local interference. Speed appears limited by friction at bends of radius <30 m and force on bends >30 m [2], and as range of radius here is calculated as 20.44 to >120 m it is likely that friction limits may play a role in limiting speed on some parts of the curve (Fig 2).

Swing duration showed a small but significant reduction of 0.96 ms (p < 0.001) per 1 m s^-1^ increase in speed. Previous studies have indicated that swing duration is constant across a range of speeds [25], likely due to a partially passive mechanism for limb protraction [28], although training may lead to decreased protraction duration under some circumstances. The change found here is statistically significant, but the biological effect in comparison to the much larger change in stance duration is likely to be extremely small.

The increase in limb angle with increasing curve and increasing speed is in agreement with findings in goats [29], which lean more with increasing speed, but not dogs, which do not necessarily increase lean consistently as speed increases [14], although the dogs in that study were not always moving at a steady speed. The inside forelimb had a greater inclination angle than the outside forelimb, which cannot be completely explained by the inside forelimb negotiating a smaller curve than the right. The average chest width of adult Thoroughbred horses is 0.42 m measured between the points of the shoulder [30]. Based on calculated predicted angles derived solely from curve radius and speed, on a curve of radius 50 m for a horse traveling at 10 m s^-1^, this would result in the inside limb having an inclination of only 0.1° greater than the outside limb and for a horse travelling at 15 m s^-1^, the inside limb would be expected to have an inclination of 0.2° greater than the outside limb (supplementary information). Limb lean was also found to be present on the straight. As data for all straight strides were pooled, it is possible that some strides represent a slight lean as the horse leaves the curve or anticipates the next curve. This study showed that predicted body lean angles underestimate MCIII angle for the inside leg when horses are galloping on the straight, and overestimate outside leg inclination angle on a curve. Predicted leans show some variation from true body lean in horses moving on circles [19], which may be explained by horses bending, by individual differences [19] or by subclinical lameness [31]. Greater differences between inside and outside limb inclination than would be predicted simply by calculating the curve radius of each limb are seen in both this study and in the study which demonstrated that on a 10 m flat curve the inside limb was at a 5.2° greater angle than the outside limb [4].

During stance, moments about the long axis of the limb cause adduction of the outside limb and abduction of the inside limb [16], which may contribute to the differences in angle observed here assuming that turning mechanisms are similar between gaits. However, it should be noted that in this study, the curve radius was much larger, and therefore different mechanisms may be used. The inside and outside limbs have different functions in the negotiation of a curve and likely experience different constraints [11]. In humans [11] and in horses [16] the outside limb is responsible for generating more vertical and lateral force than the inside limb. This is supported by anatomical data showing increased thickness of the left femur compared to the right in horses which train in a clockwise direction [32].

The only condition for which there was a difference between whole limb angle and MCIII angle was for the inside limb on a curve, with whole limb angle 1.9° greater than MCIII angle. Thoroughbreds typically show a mild carpal valgus of 5.8° standing and 5.3° when walking [33], but no conformational measurements were made in this study. However, as no differences were seen between whole limb and MCIII angle for other circumstances, it is reasonable to suggest that this difference is due to asymmetric loading about the long axis of the limb during curve running. Carpal injury in the Thoroughbred occurs most frequently in the middle carpal joint in the third and radiocarpal bones [34,35], with the most common ligamentous injury in the carpus being the medial palmar intercarpal joint [36]. There appears to be no laterality to ligament injuries in the carpus in horses training and racing on oval tracks [36]. However, when considering bony injury, 67% of third carpal bone fractures occur in the right forelimb in racehorses in the USA [37]–the outside limb when training anticlockwise. While there is evidence for uneven loading in the inside limb on a curve presented here, it is not known if it is clinically significant.

While limb angles follow predicted patterns, and limb angles were only measured when the horse appeared to be directly facing the cameras, the tracking of limbs in two dimensions can lead to error if the horse is not directly facing the camera [38]. There is also the potential for rider interference to affect lean angle, as for some trials the riders appeared to be pulling the horse’s head to the outside of the curve, presumably for control. This may explain some of the negative angles measured.

Curve running is complex and not yet fully understood [11]. There is insufficient evidence in this study does to support a force limit on performance at training speeds on larger curves, and it is likely that in this case speed was limited by rider factors, and, at higher speeds on the tighter parts of the bend, friction (Fig 2). Remodeling a racecourse to reduce curve radius [39,40] or increasing banking angle [41] significantly reduces injury rates. This is likely to be partially due to a reduction in the increased effective bodyweight–and therefore vGRF- caused by an increased centripetal force [2]. However, other factors likely play a role in limiting performance and increasing injury, as moving on a circle increases hGRF to up to 24% of vGRF [16] and work in humans has suggested that muscles stabilising joints in the sagittal plane may limit performance [11]. While muscle mass is greatly reduced in the distal limb and motion is restricted to the sagittal plane by joints rather than muscles in horses, this resistance to non-sagittal forces may also be important in the horse. This warrants further study.

This study does not take into account the hindlimbs and therefore does not account for the differing functions of the fore and hind limbs. In horses the hindlimbs mainly act to propel the centre of mass forwards [42], while the forelimbs apply vertical impulse to the centre of mass [43]. This is also likely to be the case for curve running in the horse, as other quadrupeds such as greyhounds [13] and mice [1] show similarly differing roles for the fore and hindlimbs during curve running. Further work should take into account the role of the hindlimbs and bend along the long axis of the body when galloping at high speeds on curves representative of those used in training. Additionally, a more comprehensive understanding of the extent and effects of torque about the long axis of the limb would aid in understanding injury mechanisms during curve running.

## Supporting information

S1 Data Basic data curve running. (CSV)
